# Lessons Learned From the Implementation of a Pragmatic Trial Evaluating Non‐Pharmacological Approaches to Dementia Care in US Nursing Homes

**DOI:** 10.1111/jep.70451

**Published:** 2026-04-23

**Authors:** Carin Wong, Catherine V. Piersol, Felicia Chew, Cara Lekovitch, Victoria Shier, Mike Morris, Jenny Martínez, Julie Britton, Natalie E. Leland

**Affiliations:** ^1^ Department of Occupational Therapy, School of Health and Rehabilitation Sciences University of Pittsburgh Pittsburgh Pennsylvania USA; ^2^ Department of Occupational Therapy, College of Rehabilitation Sciences Thomas Jefferson University Philadelphia Pennsylvania USA; ^3^ The Leonard D. Schaeffer Center for Health Policy & Economics, Sol Price School of Public Policy University of Southern California Los Angeles California USA; ^4^ Study Advisory Committee Member Pittsburgh Pennslyvania USA; ^5^ College of Population Health Thomas Jefferson University Philadelphia Pennsylvania USA; ^6^ Genesis HealthCare Kennett Square Pennsylvania USA

**Keywords:** community partners, implementation, lessons learned, nursing home, pragmatic trials

## Abstract

**Rationale:**

The consistent delivery of effective evidence into nursing home practice has been shown to enhance quality of care and residents' outcomes. Pragmatic trials enable the evaluation of efficacious interventions in a real‐world context, including care of residents living with Alzheimer's Disease and Related Dementias (ADRD). Yet, there are limited pragmatic trials conducted in nursing homes and even less evidence on the strategies to successfully conduct such research in this setting.

**Objective:**

To identify the lessons learned from a pragmatic trial and provide recommendations for future successful studies in United States (US) nursing homes.

**Methods:**

Drawing on a case example of a recently completed pragmatic trial, we used the Comparative Effectiveness Research Framework to guide the thematic analysis of study documentation to identify challenges, strategies used, and recommendations for future pragmatic trials in US nursing homes.

**Results:**

Five themes emerged from this case example: 1) establish and sustain community partner collaboration, 2) context analysis and appropriate implementation plan, 3) study adaptability, 4) data collection in a ‘real world’ setting, and 5) broad dissemination. Within each theme, strategies we used, challenges we encountered, and recommendations for future studies were identified. Examples of recommendations include empowering community partners to be engaged in the research, co‐design with community partners, account for the dynamic nature of the nursing home setting, and maintain communication with organization leaders.

**Conclusions:**

Successful completion of pragmatic trials in US nursing homes requires active engagement of community partners throughout the research process. The emergent lessons learned and recommendations highlight the complex nature of this care setting, the need to be proactive in implementation planning, and the pivotal role of community partners. Moving forward, there is a need for greater emphasis on systematically sharing experiences more broadly to advance the science of pragmatic trials in US nursing homes.

## 1 Introduction

Pragmatic trials are often used to evaluate real‐world intervention [1]. There is a growing interest in using this approach in long term care settings (e.g., United States (US) nursing homes) to accelerate the translation of evidence into practice [2, 3]. Pragmatic trials have been prioritized as an important methodology for the nursing home care context [4, 5].

A broad range of community partners (e.g., researchers, industry leaders) have voiced the need for pragmatic trials in nursing homes. For example, the US National Institute on Aging (NIA) funded a conference panel, which brought together researchers, nursing home leadership, and directors of professional and advocacy organizations. Discussions emphasized the importance of research in the real‐world while highlighting the challenges associated with conducting pragmatic trials in nursing homes [3, 5, 6]. The challenges described included (a) the (mis)alignment between interventions and staff workflow and organization goals; (b) the need to balance data collection burden with scientific value and relevance to the end user; and (c) getting and sustaining buy‐in from staff and leadership in light of changing priorities and turnover [3, 5, 6]. Beyond staff‐facing challenges, variability across nursing home state regulations and environmental barriers were also described as challenges that needed to be accounted for in study design [7, 8].

One population in need of evidence documenting intervention effectiveness is long term care nursing home residents living with Alzheimer's Disease and Related Dementias (ADRD). Evidence reveals disparate access to high quality US nursing homes for residents with ADRD from historically minoritized communities, variability in care quality, and disparities in health and quality of life outcomes [9, 10, 11, 12, 13, 14]. Further, staff have articulated a need for more dementia‐specific training to meet the needs of this resident population, such as managing behavioral symptoms associated with the disease process [15, 16, 17, 18, 19].

Efforts to systematically address these evidence gaps in ADRD care quality, disparities in resident outcomes, and staff training are challenged by the necessity of navigating the complexity of the nursing home care environment [5]. More specifically, US nursing home operations are influenced by federal, state, and regional policies and regulations governing the mix of patients, including short stay post‐acute care patients with a goal of returning home, long‐ term care residents that live in the facility, short term respite patients, and hospice patients at the end of life [6]. Yet, despite these varied populations and distinct policies dictating care, the same staff member supports daily operations and provides care across the entire range of patient mix. To meet these complex requirements, nursing home staff reflect a broad range of job roles, varied backgrounds and training, and levels of prior education [5].

The varied medical needs of residents and the broad range of staff need to be considered when completing pragmatic trials in the nursing home setting. Moreover, the value of strategic engagement of community partners throughout the research process (e.g., topic prioritization, study design, execution, dissemination) is essential [20, 21, 22, 23]. As such, given the complexity of the nursing home setting, the study team should consist of community partners and academic researchers that will collaboratively develop the implementation plan with the intent to deconstruct the dynamic care processes and interactions across staff within the organizational context [4, 5]. Given the need to accelerate evidence into practice, there is a need to disseminate experiences of study teams that have successfully conducted pragmatic trials in this setting. Therefore, the purpose of this paper is to identify lessons learned from the successful completion of a US nursing home based pragmatic trial and provide recommendations for future pragmatic trials.

## 2 Methods

We present a case example of a pragmatic trial, which examined two leading nonpharmacological approaches to delivering dementia care (team and problem‐based) [24, 25, 26, 27, 28]. Despite evidence supporting both approaches, there was a need to examine (a) the comparative effectiveness of the two approaches to dementia care on resident outcomes and (b) how these non‐pharmacological approaches compare after the emergence of COVID‐19. Details of the pragmatic trial methodology are published elsewhere [29]. Throughout the study process, we navigated a dynamic nursing home setting which brought along many challenges (e.g., staffing turnover, shifting clinical priorities, Covid‐19 pandemic).

### 2.1 Conceptual Framework

Our parent study was co‐designed and executed in collaboration with community partners, which involved meaningful engagement by our partners throughout every aspect of the research process. Our collaborative approach was guided by the ten steps of the Comparative Effectiveness Research (CER) framework, which provides a structure for ensuring meaningful engagement in all research activities. More specifically, it indicates academic researchers should work collaboratively with community partners through each step of the research process, including topic solicitation and prioritization, framing the question, selection of comparators and outcomes, creation of a study conceptual framework, analysis plan, data collection, reviewing and interpreting results, translation, and dissemination [22, 30, 31, 32, 33]. For this paper, we used the CER framework to examine the strategies we used, challenges we encountered, and recommendations to overcome the challenges in each step of the research process, and conduct a thematic analysis of study documentation and study team members' perspectives.

### 2.2 Data Sample

Our data sample consisted of documentation from the parent study; specifically, the structured meeting minutes from five types of meetings over the 6 years of the project period: (1) standing monthly meetings with our Advisory Committee, (2) small group Advisory Committee member meetings focused on specific actions that emerged from the larger monthly meeting, (3) weekly leadership meetings, (4) bi‐weekly clinical operations meetings, (5) bi‐weekly research focused meetings, and (6) twice yearly survey results summarizing Advisory Committee appraisal of our efforts to meaningfully engage them, including our strengths, weaknesses, areas of improvement across research activities. All meetings were held via a web‐based conference platform. Our data sample included documentation from a total of 366 meetings over the study period.

Regardless of the type of meeting, each had a structured agenda, which included standing agenda items (e.g., project status updates relevant to the meeting attendees) and emerging challenges that needed discussion. Following the meeting, detailed meeting minutes included the following components: the discussion on each agenda item, decisions made, recommended actions and responsible party, options for navigating challenges, and next step(s). Further, twice a year, in the month following the Advisory Committees' evaluation of our engagement efforts, survey results were presented at the monthly standing Advisory committee meeting. During that meeting, NL and JM would solicit feedback from the community partners on proposed strategies, as well as alternative approaches, to address weaknesses identified in the prior month's survey [31].

The perspectives from the entire team were captured in the minutes, including Advisory Committee members and the study team leaders who managed the day‐to‐day operations. As a collective group, Advisory Committee members were regularly asked to share their insights into the project (e.g., engagement efforts, study operations), including what worked well, what could have been done differently, and the lessons they learned individually as well as collectively as a group. These insights were captured via meeting minutes documentation. Study team leaders met to reflect on the project, providing a review of the study history and evolution as well as their perspectives on the challenges, actions taken, strengths, and individual recommendations. These reflections were documented by the project coordinator, who captured specific insights and reflections.

### 2.3 Data Analysis

We used a thematic analysis approach to evaluate the study documentation [34]. The coding team, which included the parent study's principal investigator (NL), co‐investigator (CP), and research team member (CW), reviewed and coded the study documentation. These codes were discussed and revised until we reached a consensus on distinct themes. Once consensus was reached, we did a member checking session with our Advisory Committee to ensure they agreed with the themes. Through this process of reviewing study documentation and team perspectives, emergent themes were identified to describe lessons learned, which included descriptions of our experiences (i.e., challenges we faced, strategies used) and informed recommendations for future studies. These emergent themes were reflective of the steps in the CER framework.

## 3 Results

Five overarching lessons learned themes emerged: 1) establish and sustain community partner collaboration, 2) context analysis and appropriate implementation plan, 3) study adaptability, 4) data collection in a “real world” setting, and 5) broad dissemination. For each lesson learned theme, we describe our experiences (i.e., strategies employed, and challenges encountered) and offer recommendations for future studies, stratified by lessons learned theme. Table 1 provides a summary of our findings and the alignment with the CER framework.

**Table 1:** Mapping experiences and recommendations for each theme to the Comparative Effectiveness Research (CER) framework.

Theme	Step(s) in comparative effectiveness framework	Experiences		Recommendations for future research
		Strategies employed	Challenges encountered	
Establish and sustain community partner collaboration	Not reflected in the CER framework.	Developed academic‐clinical partnership. ⚬ Worked with community partners to identify clinical priorities. ⚬ Worked with community partners to identify and pursue funding opportunities (e.g., collaborate on writing grant proposals). Established a shared governance with Advisory Committee. Engaged Advisory Committee and sought their feedback on all aspects of the study throughout the process. Regularly sought the Advisory Committee's perspective on researchers' efforts to meaningfully engage them via systematic evaluation.	Some members of the research team had not previously worked collaboratively with community partners.	Co‐create a governance structure with engagement of both academic researchers and community partners. Acknowledge and adapt meeting schedule to community partners' schedules and obligations. Assess and address research team's readiness to work with community partners.
Context analysis and appropriate implementation plan	Creation of conceptual framework	Used conceptual frameworks to guide the implementation plan of the intervention. Considered variation in organizational structures. Maintained open lines of communication with Advisory Committee to address emerging implementation challenges associated with the complexity of the setting.	Did not account for policies and regulations for other nursing home patient populations. Needed to consider the nursing home sample and the potential impact the dynamic nursing home setting had on continued participation in the study.	Plan and account for the dynamic nature of the nursing home context by using implementation frameworks to help with study design. Work with community partners to understand the nursing home context during implementation planning and throughout the study process to address emergent challenges. Oversample nursing homes to account for attrition.
Study adaptability	Data collection	Strategies	Did not consider electronic data collection for pre/post test data.	Proactively deconstruct the study components within the context of the nursing home to understand which components can be adapted. Leverage technology when collecting prospective data.
		Considered the differences in nursing homes and considered which components of the study needed to be adapted. Communicated with Advisory Committee on strategies to adapt intervention.		
Data collection in a ‘real world’ setting	Data collection	Integrated study data collection needs into staff workflow. Collaborated with the systems information technology (IT) specialist to find the best way to obtain data.	Did not sufficiently consider the different resources and IT capabilities of each nursing home to extract Centers for Medicare and Medicaid Services (CMS) required Minimum Data Set (MDS) data for the study team. Organizations had different formats for providing the MDS data to the study team	Integrate data collection within the staff's normal work routine. Work with content expert and systems IT representatives as community partners throughout the study process. Build in time for understanding the different IT capabilities of participating nursing homes, even when relying on CMS required fields as study data points. Build in time for understanding the different formats that data may be received from various participating nursing homes and preparing raw data for analysis.
Broad dissemination	Translation Dissemination	Considered what was meaningful to the researchers and nursing home community when developing and executing the dissemination plan. Engaged in an ongoing discussion with the Advisory Committee about the dissemination plan throughout the study.	Advisory Committee lacked self‐efficacy to contribute to traditional academic products.	Create and implement the dissemination plan with community partners to engage in dissemination activities throughout the research process. Empower Advisory Committee participation in dissemination activities, conveying the value of their contribution(s) to dissemination products.

### 3.1 Theme #1: Establish and Sustain Community Partner Collaboration

Meaningful community engagement from idea prioritization, co‐design, implementation, and dissemination was pivotal to our study success. Establishing and sustaining community partner collaboration throughout the entire research process was not a distinct step in the CER framework. We worked collaboratively with our community partners throughout the research process, which spanned idea prioritization and review of existing evidence, to study implementation and dissemination of study practices and results. Our community partners included representatives from all aspects of nursing home care such as resident advocates, family caregivers, registered nurses, certified nursing assistants, social worker, dietary staff, physical therapists, occupational therapists, administrators, etc.

**Figure 1:**
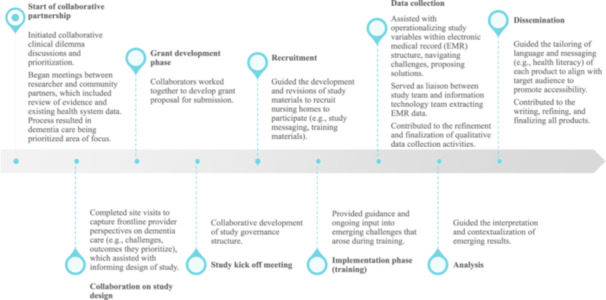
Examples of community partners' meaningful engagement throughout the study process.

We used co‐design principles, which uses a collaborative approach of including end users as actual designers of a study [30]. These co‐design principles were guided by the Patient‐Centered Outcomes Research Institute Engagement Rubric [35]. Figure 1 highlights examples of engagement from our community partners at various phases of the study.

#### 3.1.1 Strategies Employed

##### 3.1.1.1 Facilitate Idea Prioritization

Prior to idea conceptualization for the parent study, a partnership between academic researchers and a nursing home organization leader was formed and nurtured. These partners worked together over time to understand and prioritize health system clinical dilemmas, review existing data, and evaluate evidence, which led to dementia care being prioritized as the clinical dilemma that would be addressed in their partnership. In order to understand this clinical dilemma from multiple perspectives of the nursing home community, we pursued the insights of frontline providers (e.g., certified nursing assistants, social services, rehabilitation providers, etc.) delivering dementia care, including the challenges they experienced and their recommendations for outcomes we should include in the study proposal. In addition to the conversations we had with frontline providers, we also met separately with nursing home leadership to gather their perspectives and recommendations. The partners collectively further refined the clinical dilemma, which produced a final study proposal that focused on examining two evidence‐based nonpharmacological approaches to dementia care. These meetings with members of the nursing home community helped inform the design, scope, and framing of the study detailed in the resulting grant proposal. Through this academic‐clinical partnership, we worked together to identify the funding mechanism that fit our collective priorities and collaboratively write the grant proposal. A nursing home organization leader was partnered as a Principal‐Investigator (PI) on the multi‐PI grant. The co‐development of the grant included idea prioritization, reviewing the evidence, and implementing co‐design principles [36, 37].

##### 3.1.1.2 Foster Collaboration

When the study began, we made active efforts to ensure our Advisory Committee members were meaningfully engaged in all aspects of the study. Our efforts were guided by established engagement principles, including fostering reciprocal relationships, co‐learning, partnerships, transparency, honesty and trust [38]. We worked with our community partners to formalize our collaboration through the development of an engagement charter, which we co‐created together [32]. The charter was used to establish a shared governance and ensure equity so that all members of the team (i.e. academic researchers, community partners) understood their roles, responsibilities, and expectations. The charter also formalized methods and expectations for communication, established processes for shared decision‐making, and created a structure for members to rotate off the Advisory Committee.

##### 3.1.1.3 Engage Study Team

Throughout the study, we continued working with our community partners via monthly meetings and, as needed, small group task‐directed meetings [30, 36, 37]. For example, during the initial nursing home recruitment stage, they guided the development of messaging about the study, reviewed and provided direction on the refinement of the recruitment materials, and provided suggestions on how to refine the process of engaging facilities to participate. During the planning of the training phase of the study, Advisory Committee members were pivotal in providing strategies to assist with staff receiving the intervention, such as insights on facility operations. Further, they also provided guidance and contributed their various expertise (e.g., frontline staff, leadership informal family caregiver) in our efforts to contextualize and interpret preliminary analysis, by offering additional considerations on the interpretation of the qualitative data.

##### 3.1.1.4 Offer Consistent Communication

We were consistent in our scheduling of monthly Advisory Committee meetings, which were done a year in advance. Throughout the duration of our study, we offered three standing meeting times each month. Of which, each Advisory Committee member could select which of those three meetings to participate in each month. These meeting times accounted for the multiple time zones represented among members and were determined by the Advisory Committee at the start of the project. Agendas for a given month were distributed to all members a minimum of one week in advance. Monthly meeting minutes from each of the three sessions were accessible to all members to review to promote transparency across sessions and members. Finally, we were intentional when requesting their participation. If there were no topics to be discussed in a given month, the meetings were cancelled for that month.

##### 3.1.1.5 Evaluation of Engagement Efforts

In order to ensure our community partners were meaningfully engaged in the study, complying with established engagement principles, we regularly evaluated our engagement efforts [31]. Every six months, our Advisory Committee completed an evaluation survey that assessed the study teams' engagement strengths, weaknesses, and areas in need of improvements, which spanned across the engagement principles (e.g., co‐learning, partnership). Once the evaluation survey was completed, the results were reviewed by study leadership to identify what was done well, where we fell short, and develop our plans to improve. Within 30‐days, results and the plan for remediation were presented to the Advisory Committee to promote transparency and seek input on the action plan, results of this process have been presented elsewhere [31].

##### 3.1.1.6 Retain Advisory Committee

We were able to maintain a strong commitment throughout the entire study, as reflected by our retention of Advisory Committee members. One member requested to rotate off the committee due to unavoidable life circumstances, which were not related to the study. Our engagement scores from our evaluation survey consistently increased over time [31].

#### 3.1.2 Challenges Encountered

##### 3.1.2.1 Research Team Knowledge and Experience

We proactively anticipated and planned to equip our community partners with knowledge about the research process. Our intent was not to produce research experts, but ensure our community partners had a foundational understanding of the methods in order to engage in discussion about the study. We found that some members of the study team had not previously engaged in research with community partner collaboration, only as research participants. As a result, early in the project, these individuals had difficulty working with the Advisory Committee as partners engaged in shared decision making, as we had co‐created our governance structure. There were opportunities to better equip these study team members with knowledge and skills for collaboration with community partners.

#### 3.1.3 Recommendations

##### 3.1.3.1 Foster a Collaborative Partnership with Community Members

We highlight the importance of co‐creating a governance structure with the Advisory Committee so that there are clear roles, responsibilities, and expectations for all parties. All community partners were financially compensated equally, which included receiving the same stipends for each hour of engagement, regardless of their job title (e.g., physician, nurse, caregiver, etc.). This approach was used to create an equitable environment where everyone's perspectives were respected and valued.

##### 3.1.3.2 Acknowledge the Differing Schedules of Study Team

To foster engagement from the Advisory Committee, we encourage teams to establish standing meetings well in advance while providing flexibility that accounts for the range of time zones represented. This approach will account for their busy schedules and emergent obligations.

##### 3.1.3.3 Address the Readiness of the Study Team

One strategy that we did not implement but wish we had was to assess and address the study team's readiness to work with community partners. This approach could have mitigated the challenge we experienced due to some team members' lack of experience partnering with the community to collaboratively conduct research. Establishing a baseline level of readiness and addressing the need for knowledge and strategies on how to work in a team‐setting with a broad range of individuals would have been beneficial.

### 3.2 Theme #2: Context Analysis and Appropriate Implementation Plan

Nursing homes are dynamic care settings. There are various factors that may be impacting the day‐to‐day organizational operations, such as federal, state, and local policies, as well as different care priorities, and leadership turnover. Further, each nursing home may have different experiences based on setting‐specific circumstances. Thus, when designing and executing pragmatic trials, these factors need to be considered, particularly as it relates to the context analysis and designing the intervention implementation plan, which are reflected in the CER Framework under ‘creation of the study conceptual framework’ step.

#### 3.2.1 Strategies Employed

##### 3.2.1.1 Apply Conceptual Frameworks

Conceptual frameworks can be used to help guide the implementation of the intervention. These frameworks include the processes and procedures for the intervention, the individuals involved, and the context related to the implementation (e.g., organizational context). In our pragmatic trial, we used two conceptual frameworks to guide our study. First, we used the Health Equity Implementation Framework to inform sampling of US nursing homes (e.g., inclusion, exclusion) and study design (e.g., intervention implementation) to ensure our study did not exclude facilities that provided care to residents that historically faced inequities in dementia care and the design accounted for variation in nursing home contexts [39]. The second framework was Donabedian's 3‐component model of quality [40]. This framework was used to examine the relationship between the study interventions and the outcomes of long‐stay residents with dementia.

##### 3.2.1.2 Considered Variation in Organizational Structures

We used the conceptual frameworks to help understand the differences in the organizational structures, allowing for us to account for the variation across study sites. For example, using the Health Equity Implementation Framework, it helped inform our sampling and consider the disparities in access to high quality nursing homes among historically underrepresented populations of older adults.

##### 3.2.1.3 Maintain Open Lines of Communication

To address the variation in organizational structures across all participating nursing homes, we worked with our Advisory Committee to address emergent challenges that arose that related to the complexity of the organization and uncertainty of the nursing home industry (e.g., challenges they faced during the COVID‐19 pandemic). We gained additional insight from Advisory Committee members that had firsthand knowledge of the nursing home setting.

#### 3.2.2 Challenges Encountered

##### 3.2.2.1 Nursing Home Policies and Regulations

In the study implementation planning phase, we analyzed the context of the nursing home setting. Throughout this process, we focused on the long‐term care residents with dementia as that was the population our study was evaluating. We did not account for the short‐stay nursing home patients that were receiving care in the participating US nursing homes. As a result, our planning process did not account for the potential impact of policies and regulations that were being implemented across the United States, targeting this other nursing home population. For example, in October 2019, more than a year after our study had started, the Patient Driven Payment Model (PDPM) was implemented. This was a change to Medicare Part A payment policy, which updated how nursing homes were paid for their short‐stay Medicare patients. While not directly related to long‐term residents, which were the focus of our study, the staff that had to learn about and implement this new payment policy were the same staff who also supported the daily operations and care of the long‐term care residents living with ADRD. As a result, the implementation of this short‐stay patient policy made it a challenge to engage staff in intervention implementation training at this time, affecting the timeline of recruitment and extending the implementation window of the study intervention.

##### 3.2.2.2 Dynamic Nature of the Nursing Home Setting

Another factor that we did not account for was the overall dynamic nature of the nursing home healthcare setting. Given the duration of our study, the nursing home facilities in our trial underwent leadership turnover, changing priorities of the organization, and the emergence and evolution of the Covid‐19 pandemic. These were unanticipated factors that impacted the recruitment and retention of facilities, which decreased the sample size of our study.

#### 3.2.3 Recommendations

##### 3.2.3.1 Plan and Account for the Dynamic Nature of the Nursing Home Setting

First, be intentional in the use of an implementation framework in the design of the study. This allows us to thoughtfully plan out the study design, including sampling frame and implementation plan to ensure we consider the multiple levels of context (e.g. external environment, organization), all of which impacts study success.

##### 3.2.3.2 Work With Community Partners

Throughout the study, work with community partners to understand and consider factors that impact the nursing home, beyond those specific to the patient population that is the focus of the study (e.g., long‐term and short‐term patients).

##### 3.2.3.3 Oversample Nursing Homes

Given the dynamic nature of the healthcare setting and the possibility of losing facility participants due to these factors, consider oversampling of nursing homes to account for attrition due to unanticipated factors.

### 3.3 Theme #3: Study Adaptability

When developing a study, we considered the components of the study that could be adapted and/or tailored for the individual nursing home context, which is reflected in the CER framework. Given the variability in nursing home settings, this includes embedding flexibility in the implementation of the intervention.

#### 3.3.1 Strategies Employed

##### 3.3.1.1 Adapt/Tailor the Intervention

During the design of the study, we considered various strategies to adapt the intervention to the differences in structures and processes across nursing homes [29]. For example, our implementation plan accounted for nursing homes that had paper‐based documentation and those that were using electronic medical records. We identified strategies to integrate the intervention into the staff workflow based on their documentation structure (i.e., paper, electronic).

##### 3.3.1.2 Communicate With Advisory Committee for Intervention Adapting Strategies

An example of adaptation is addressing the variations in the physical structures of nursing homes. There were instances when we had to provide alternative strategies to intervention components due to differences in the building structure of different nursing homes. For example, our intervention relied on visual images being posted on the doorway of resident's rooms. These visual cues conveyed the resident's stage of dementia and served as indicators for staff. The initial intervention placed magnets on residents' room doorways. However, our implementation plan had to account for older buildings that had wooden doorways. As a result, we adapted this part of the intervention by providing alternative approaches to posting visual images in the resident's room to cue staff when doorway magnet placement was not feasible. To come up with alternative strategies, we worked with our Advisory Committee to identify other methods for posting signage to indicate to staff the residents' cognitive stage.

#### 3.3.2 Challenges Encountered

##### 3.3.2.1 Consideration of Electronic Data Collection

A challenge we experienced as part of our intervention related to data collection from the staff training. As part of our study, we requested staff to complete a pre‐ and post‐test to assess their knowledge in dementia care before and after completing their training modules. The pre‐test was completed when our trainers were in the facility and were able to collect the tests in‐person. After completing the final module, facilities were to return the post‐tests via postal mail with pre‐paid shipping materials, which were provided by the study team. With the receipt of the returned post‐tests, the study team would provide the facility with reimbursement for the staff time engaging in the training. However, this procedure resulted in a lower number of post‐tests being returned as compared to the pre‐tests, which impacted our results. If we were to do this again, we would consider capturing this information electronically, particularly considering the evolution of technology, which could have helped increase staff participation.

#### 3.3.3 Recommendations

##### 3.3.3.1 Proactively Deconstruct Study Intervention Components

Invest time upfront to proactively deconstruct the study intervention components within the context of the nursing home and consider the variability in organizational processes as well as the physical, regulatory, and social context. Given these contextual factors, there are certain intervention components that may need to be tailored to foster success across nursing homes.

##### 3.3.3.2 Leverage Technology for Data Collection

Consider leveraging the most up to date technology for data collection when capturing information outside the standard staff workflow, which may assist with prospective data collection.

### 3.4 Theme #4: Data Collection in a ‘Real World’ Setting

When determining the methods we planned to use and variables included in the study within a ‘real world’ setting, we considered the impact that data collection would have on the staff. We developed our data collection plan to limit disruption to the staff workday. This was a step highlighted in the “data collection” step of the research process from the CER Framework.

#### 3.4.1 Strategies Employed

##### 3.4.1.1 Balance Data Collection Burden

We collaborated with community partners to co‐design the optimal strategy for data collection, which provided meaningful research data while minimizing the burden on staff. Our data collection plan weighed the staff burden associated with prospective data collection outside mandatory clinical care reporting with opportunities for leveraging data already collected as part of staff daily workflow. For example, our community partners encouraged us to use the Minimum Data Set (MDS) residential assessment instrument as our source of data to capture resident‐level data (e.g., sociodemographic characteristic, medical status, and outcomes). This approach was chosen because the Centers for Medicare and Medicaid Services requires nursing homes to collect and submit MDS data on a regular basis for all residents and would not add an additional data collection burden to staff within their daily workflow.

##### 3.4.1.2 Collaborate With the Systems Information Technology Specialist

To monitor staff burden and workflow, we were in regular communication with the staff to gauge any interruption in their workflow. We considered strategies that could be implemented to reduce the burden on the staff. For example, when we were trying to obtain MDS data, we collaborated with the systems information technology (IT) specialist, who was part of our Advisory Committee, to identify the best way to obtain the data.

#### 3.4.2 Challenges Encountered

##### 3.4.2.1 Different Resources and IT Capabilities of Nursing Homes

We had initially decided to use MDS data to limit the request for staff to do more within their daily responsibilities by requiring them to contribute to additional data collection. We felt this was the best approach as all US nursing homes that are certified by the Centers for Medicare and Medicaid Services are required to complete the MDS for their residents on a regular basis, creating common data elements across participating sites. However, there were factors that we did not account for. During data extraction of MDS data, we found wide variability in resources that nursing homes had available to pull the data. Specifically, nursing homes varied in terms of IT support to extract MDS data for research. Some facilities had a data warehouse and an IT specialist on the staff to assist with data extraction. Meanwhile, there were other nursing homes that did not have IT capabilities, so we had to contact their software provider company directly to learn how their system worked. Once we understood their system, we then worked with the nursing home staff to implement how to extract the appropriate data.

##### 3.4.2.2 Different Data Formats

Even though the MDS is a mandated and standardized assessment, the format of the data that we received varied widely across the different organizations. As a result, we had to spend additional time preparing the data for analysis.

#### 3.4.3 Recommendations

##### 3.4.3.1 Integrate Data Collection Into Staff Work Routine

When designing the data collection plan, prioritize minimizing the burden on nursing home staff and limit disruptions in their workflow. We found that it was important to embed data collection within the staff's normal work routine (e.g., capitalizing on required data fields in MDS data), to decrease disruptions, limit risk of missing data, and optimize data completeness.

##### 3.4.3.2 Work With Content Expert and Systems IT Representatives

Communicate with organization leaders early and often to understand their IT capabilities for extracting data from their system. Even when efforts are made to use standardized and mandated data, there may still be variability across organization in the resources they have available to extract their data as well as the format of that data. It is also important to include both content experts and systems IT representatives on Advisory Committees to help better understand the different ways that data can be extracted and received from different facilities.

##### 3.4.3.3 Build in Time for Data Collection and Analysis

Research teams should consider building in additional time (a) on the front end to understand the data format and resources available for extracting data from participating organizations and (b) on the back end when preparing for data analysis.

### 3.5 Theme #5: Broad Dissemination

Dissemination is the final step in the CER framework. When disseminating research, we navigated the competing priorities of academic researchers and community partners.

#### 3.5.1 Strategies Employed

##### 3.5.1.1 Employ Meaningful Forms of Dissemination

To maintain an equitable partnership, we considered what was meaningful to the researchers and the nursing home community when creating our disseminating plan. For example, we worked with our Advisory Committee to identify the types of materials they would find meaningful in their various nursing home community roles. They guided the development and implementation of a dissemination plan that went beyond traditional academic peer review journals and scientific presentations. Based on their recommendations, we developed a series of dissemination materials that targeted nursing home staff, residents, and family members (e.g., one‐page flyers on various study‐specific topics).

##### 3.5.1.2 Work With Advisory Committee to Develop Dissemination Plan

We engaged in dissemination activities throughout the study and consistently included members of the Advisory Committee in each of the products when targeting the research community. For example, in the early stages of the study, we shared knowledge about our community engagement methods and tools we developed to help other researchers. We also participated in presentations at national conferences and developed manuscripts, which helped reach other researchers in the field.

#### 3.5.2 Challenges Encountered

##### 3.5.2.1 Advisory Committee Members' Self‐Efficacy

During the dissemination process, we tried to engage Advisory Committee members in the development of different dissemination products as co‐authors. However, many of the Advisory Committee members articulated not feeling that they could provide meaningful contribution to traditional academic products (e.g., peer reviewed manuscripts, national conference presentations). They were more comfortable contributing to the development of materials that were provided to the nursing home community (e.g., leaders, staff, and family caregivers). To overcome this challenge, the first author of each paper would lay out the expectations and frame the perspective that each Advisory Committee member could provide to the paper. Further, the first author would often meet with these co‐authors one‐on‐one via web‐based conferencing to talk through the paper, seek input, and document the changes proposed by the Advisory Committee member in place of emailing the word document and asking for edits to be made via track changes.

#### 3.5.3 Recommendations

##### 3.5.3.1 Develop Dissemination Plan

Dissemination activities should occur throughout the research process, with the development of a dissemination plan starting early in the study timeline, with input from community partners.

##### 3.5.3.2 Empower All Community Partners to Contribute Their Expertise

Be aware of community partner's limited self‐efficacy in contributing to dissemination products. To address their hesitancy, the academic researchers should proactively and clearly articulate the role the community partner will play in the product, including identifying the perspective they bring to the paper, and the value of that contribution to the dissemination product(s).

## 4 Discussion

This paper analyzed study documentation and team member perspectives, including Advisory Committee members and study team leaders who participated in a large pragmatic trial conducted in US nursing homes. Five lessons emerged from this case example, which informed the recommendations that were described. We discuss the salient recommendations here that were critical to our success. These recommendations can provide guidance to future teams conducting research in this care setting by highlighting steps to consider when developing and implementing a pragmatic trial.

When designing and implementing a pragmatic trial, establishing a structure for meaningful collaboration with community partners is pivotal. Our experience aligns with recommendations from prior studies in other clinical care settings that stress the need for building community partnerships in pragmatic trials [4, 41]. Similarly, in broader literature, there has been a call for co‐design in developing and conducting pragmatic trials [36, 42, 43]. While definitions for co‐design vary, many descriptions of co‐design include the involvement of community partners throughout all aspects of the study, which reflects what we did in this study [23, 42]. Unlike other nursing home research where co‐design principles may be integrated at various stages of a study [44, 45], we created a shared governance structure with our Advisory Committee and co‐developed a charter, at the start of the funded study, to ensure that all roles and responsibilities were clear [32]. This helped support engagement and transparency between the Advisory Committee and the study team. Moreover, understanding the readiness of study team members to work with community partners is imperative to successful collaboration. Research describes the need for collaboration [36, 42], but it is also important to assess study team members and provide education and strategies to work with all members of a team.

Another critical feature for success is ensuring the capacity to adapt the intervention and be flexible during study implementation, which our community partnerships supported. Despite proactive planning guided by the study's conceptual framework, additional adaptation of intervention components was necessary due to the wide variation in nursing home contexts [29], including unaccounted barriers that are common in nursing home settings, such as unanticipated staff workflow disruptions [46, 47]. Although other studies acknowledge these factors as limitations impacting the study [46, 47], we were able to work with our Advisory Committee members to ascertain continual feedback and recommendations for how best to strategize and overcome these challenges.

We recommend being in consistent communication with organization leaders across nursing homes. This could facilitate a better understanding of the resources the organization has to put in place to support the research study (e.g., with data collection). Although not directly related to dementia care, studies conducted in nursing homes highlight the benefit of collaborating with nursing home leaders to identify what is already in place in terms of resources, staff levels, and structure to best implement the study intervention [46, 48, 49].

Finally, prioritizing the need to educate and empower community partners to be comfortable engaging in all aspects of the research process is vital. Collaborating with community partners is essential for the successful completion of pragmatic trials, which has been demonstrated in previous work [4, 41]. However, this body of work does not describe specific strategies to support community partners and how best to acknowledge the value they bring to the work. We found success in the co‐creation of dissemination products when we provided clear expectations and identified the specific value the Advisory Committee member contributions offered.

## 5 Conclusions

Engaging community partners throughout the research process is a key factor for pragmatic trial success in US nursing homes. Pragmatic trials are an efficient way to accelerate evidence into practice. Yet, given the complex nature of this care setting, this method requires systematic and proactive implementation planning, ongoing communication, and engagement with sites as well as community partners. While this paper presents lessons learned and recommendations from one trial, there needs to be a greater emphasis on systematically sharing experiences more broadly to advance the science of pragmatic trials in US nursing homes.

## Disclosure

The views in this work are solely the responsibility of the authors and do not necessarily represent the views of the Patient‐Centered Outcomes Research Institute (PCORI), its Board of Governors, or Methodology Committee.

## Conflicts of Interest

The authors declare no conflicts of interest.

## Data Availability

The data that support the findings of this study are available from the corresponding author upon reasonable request.

## References

[1] I. Ford and J. Norrie , “Pragmatic Trials,” New England Journal of Medicine 375, no. 5 (2016): 454–463. 27518663 10.1056/NEJMra1510059

[2] K. H. Magid , E. Galenbeck , and C. Levy , “How Pragmatic Are Trials in Nursing Home Settings?,” Journal of the American Medical Directors Association 21, no. 12 (2020): 1821–1823, 10.1016/j.jamda.2020.07.014.32859515

[3] C. Levy , S. Zimmerman , V. Mor , et al., “Pragmatic Trials in Long‐Term Care: Implementation and Dissemination Challenges and Opportunities,” Journal of the American Geriatrics Society 70, no. 3 (March 2022): 709–717, 10.1111/jgs.17698. 35195281 PMC8944211

[4] S. Zimmerman , B. Resnick , J. Ouslander , et al., “Pragmatic Trials and Improving Long‐Term Care: Recommendations From a National Institutes of Health Conference,” Geriatric Nursing (New York, N.Y.) 44 (March/April 2022): 288–292, 10.1016/j.gerinurse.2022.02.008. 35219536 PMC8995377

[5] B. Resnick , S. Zimmerman , J. Gaugler , et al., “Pragmatic Trials in Long‐Term Care: Research Challenges and Potential Solutions in Relation to Key Areas of Care,” Journal of the American Geriatrics Society 70, no. 3 (March 2022): 718–730, 10.1111/jgs.17699. 35195283 PMC8904288

[6] C. Drake , H. L. Wald , L. B. Eber , J. I. Trojanowski , K. A. Nearing , and R. S. Boxer , “Research Priorities in Post‐Acute and Long‐Term Care: Results of a Stakeholder Needs Assessment,” Journal of the American Medical Directors Association 20 (April 2019): 911–915, 10.1016/j.jamda.2019.02.018. 30982714

[7] D. B. Mukamel , D. L. Weimer , C. Harrington , W. D. Spector , H. Ladd , and Y. Li , “The Effect of State Regulatory Stringency on Nursing Home Quality,” Health Services Research 47, no. 5 (2012): 1791–1813. 22946859 10.1111/j.1475-6773.2012.01459.x PMC3513606

[8] H. Temkin‐Greener , N. T. Zheng , S. Cai , H. Zhao , and D. B. Mukamel , “Nursing Home Environment and Organizational Performance: Association With Deficiency Citations,” Medical Care 48, no. 4 (April 2010): 357–364, 10.1097/MLR.0b013e3181ca3d70. 20220535 PMC2925404

[9] M. Rivera‐Hernandez , A. Kumar , G. Epstein‐Lubow , and K. S. Thomas , “Disparities in Nursing Home Use and Quality Among African American, Hispanic, and White Medicare Residents With Alzheimer's Disease and Related Dementias,” Journal of Aging and Health Aug 31, no. 7 (2019): 1259–1277, 10.1177/0898264318767778. 29717902 PMC6167186

[10] D. B. Mukamel , H. Ladd , D. Saliba , and R. T. Konetzka , “Dementia, Nurse Staffing, and Health Outcomes in Nursing Homes,” Health Services Research 59 (December 2024): 14270, 10.1111/1475-6773.14270. PMC11250382 38156513

[11] D. B. Mukamel , D. Saliba , H. Ladd , and R. T. Konetzka , “Dementia Care Is Widespread in US Nursing Homes; Facilities With The Most Dementia Patients May Offer Better Care,” Health Affairs (Project Hope) 42, no. 6 (2023): 795–803, 10.1377/hlthaff.2022.01263. 37276482 PMC10796080

[12] F. W. Porell and M. Carter , “Discretionary Hospitalization of Nursing Home Residents With and Without Alzheimer's Disease: A Multilevel Analysis,” Journal of Aging and Health 17, no. 2 (April 2005): 207–238, 10.1177/0898264304274302. 15750052

[13] T. P. Shippee , R. R. Parikh , Z. G. Baker , et al., “Racial Differences in Nursing Home Quality of Life Among Residents Living With Alzheimer's Disease and Related Dementias,” Journal of Aging and Health Jun 36, no. 5/6 (2024): 379–389, 10.1177/08982643231191164. 37493607 PMC11556434

[14] T. T. Shippee , W. Ng , Y. Duan , et al., “Changes over Time in Racial/Ethnic Differences in Quality of Life for Nursing Home Residents: Patterns Within and Between Facilities,” Journal of Aging and Health 32, no. 10 (December 2020): 1498–1509, 10.1177/0898264320939006. 32648793 PMC9121738

[15] H. Fillit , M. S. Aigbogun , P. Gagnon‐Sanschagrin , et al., “Impact of Agitation in Long‐Term Care Residents With Dementia in the United States,” International Journal of Geriatric Psychiatry 36, no. 12 (December 2021): 1959–1969, 10.1002/gps.5604. 34286877 PMC9291552

[16] C. Beck , A. Ortigara , S. Mercer , and V. Shue , “Enabling and Empowering Certified Nursing Assistants for Quality Dementia Care,” International Journal of Geriatric Psychiatry 14, no. 3 (March 1999): 197–211; discussion 211‐2. 10202662 10.1002/(sici)1099-1166(199903)14:3<197::aid-gps972>3.0.co;2-q

[17] G. A. Warshaw and E. J. Bragg , “Preparing the Health Care Workforce to Care for Adults With Alzheimer's Disease and Related Dementias,” Health Affairs (Project Hope) 33, no. 4 (April 2014): 633–641, 10.1377/hlthaff.2013.1232. 24711325

[18] Institute of Medicine Committee on the Future Health Care Workforce for Older A ., Retooling for an Aging America: Building the Health Care Workforce (National Academies Press (US) Copyright 2008 by the National Academy of Sciences, 2008). 25009893

[19] D. Carrier , É. Toulouse , and C. M. Rochefort , “Staff Training Interventions to Prevent or Reduce Behavioural and Psychological Symptoms of Dementia in Nursing Home Residents: A Mixed Methods Systematic Review,” Dementia and Geriatric Cognitive Disorders 52, no. 3 (2023): 117–146, 10.1159/000530503. 37075737

[20] J. A. Palmer , V. A. Parker , V. Mor , et al., “Barriers and Facilitators to Implementing a Pragmatic Trial to Improve Advance Care Planning in the Nursing Home Setting,” BMC Health Services Research 19 (2019): 1–12. 31357993 10.1186/s12913-019-4309-5 PMC6664774

[21] G. Jamin , T. Luyten , R. Delsing , and S. Braun , “The Process of Co‐Creating the Interface for VENSTER, an Interactive Artwork for Nursing Home Residents With Dementia,” Disability and Rehabilitation. Assistive Technology 13, no. 8 (November 2018): 809–818, 10.1080/17483107.2017.1385102. 29037109

[22] S. B. Gesell , K. P. Klein , J. Halladay , et al., “Methods Guiding Stakeholder Engagement in Planning a Pragmatic Study on Changing Stroke Systems of Care,” Journal of Clinical and Translational Science 1, no. 2 (April 2017): 121–128, 10.1017/cts.2016.26. 28649454 PMC5471818

[23] P. Slattery , A. K. Saeri , and P. Bragge , “Research Co‐Design in Health: A Rapid Overview of Reviews,” Health Research Policy and Systems 18, no. 1 (February 2020): 17, 10.1186/s12961-020-0528-9. 32046728 PMC7014755

[24] H. C. Kales , L. N. Gitlin , and C. G. Lyketsos , Detroit Expert Panel on Assessment and Management of Neuropsychiatric Symptoms of D ., “Management of Neuropsychiatric Symptoms of Dementia in Clinical Settings: Recommendations From a Multidisciplinary Expert Panel,” Journal of the American Geriatrics Society 62, no. 4 (April 2014): 762–769, 10.1111/jgs.12730. 24635665 PMC4146407

[25] L. Chenoweth , Y. H. Jeon , J. Stein‐Parbury , et al., “PerCEN Trial Participant Perspectives on the Implementation and Outcomes of Person‐Centered Dementia Care and Environments,” International Psychogeriatrics 27, no. 12 (December 2015): 2045–2057, 10.1017/S1041610215001350. 26307245

[26] Y. H. Jeon , J. Govett , L. F. Low , et al., “Care Planning Practices for Behavioural and Psychological Symptoms of Dementia in Residential Aged Care: A Pilot of an Education Toolkit Informed by the Aged Care Funding Instrument,” Contemporary Nurse 44, no. 2 (June 2013): 156–169, 10.5172/conu.2013.44.2.156. 23869500

[27] M. Halek , M. N. Dichter , T. Quasdorf , C. Riesner , and S. Bartholomeyczik , “The Effects of Dementia Care Mapping on Nursing Home Residents' Quality of Life and Staff Attitudes: Design of the Quasi‐Experimental Study Leben‐QD II,” BMC Geriatrics 13 (2013): 53, 10.1186/1471-2318-13-53. 23725292 PMC3691737

[28] T. Quasdorf , C. Riesner , M. N. Dichter , O. Dortmann , S. Bartholomeyczik , and M. Halek , “Implementing Dementia Care Mapping to Develop Person‐Centred Care: Results of a Process Evaluation Within the Leben‐QD II Trial,” Journal of Clinical Nursing 26, no. 5/6 (March 2017): 751–765, 10.1111/jocn.13522. 27534732

[29] N. E. Leland , V. Shier , C. V. Piersol , et al., “Evaluating Non‐Pharmacological Approaches to Nursing Home Dementia Care: A Protocol,” Contemporary Clinical Trials Communications 34 (August 2023): 101161, 10.1016/j.conctc.2023.101161. 37347001 PMC10266886

[30] M. Antonini , “An Overview of Co‐Design: Advantages, Challenges and Perspectives of Users' Involvement in the Design Process,” Journal of Design Thinking 2, no. 1 (2021): 45–60.

[31] J. Martínez , C. V. Piersol , S. Holloway , L. Terhorst , and N. E. Leland , “Evaluating Stakeholder Engagement: Stakeholder‐Centric Instrumentation Process (SCIP),” Western Journal of Nursing Research 43, no. 10 (2021): 949–961, 10.1177/01939459211004274. 33896283 PMC8429065

[32] J. Martínez , C. V. Piersol , K. Lucas , and N. E. Leland , “Operationalizing Stakeholder Engagement Through the Stakeholder‐Centric Engagement Charter (SCEC),” supplement, Journal of General Internal Medicine 37, no. S1 (April 2022): 105–108, 10.1007/s11606-021-07029-4. 35349021 PMC8994015

[33] A. M. Abdulhalim , “Continuous Patient Engagement in Comparative Effectiveness Research,” Journal of the American Medical Association 307, no. 15 (April 2012): 1587–1588, 10.1001/jama.2012.442. 22511684

[34] J. Thomas and A. Harden , “Methods for the Thematic Synthesis of Qualitative Research in Systematic Reviews,” BMC Medical Research Methodology 8 (July 2008): 45, 10.1186/1471-2288-8-45. 18616818 PMC2478656

[35] S. Sheridan , S. Schrandt , L. Forsythe , T. S. Hilliard , and K. A. Paez , “The PCORI Engagement Rubric: Promising Practices for Partnering in Research,” Annals of Family Medicine 15, no. 2 (March 2017): 165–170, 10.1370/afm.2042. 28289118 PMC5348236

[36] A. Zogas , K. E. Sitter , A. M. Barker , et al., “Strategies for Engaging Patients in Co‐Design of an Intervention,” Patient Education and Counseling 123 (June 2024): 108191, 10.1016/j.pec.2024.108191. 38367306

[37] C. Vargas , J. Whelan , J. Brimblecombe , and S. Allender , “Co‐Creation, Co‐Design, Co‐Production for Public Health – a Perspective on Definitions and Distinctions,” Public Health Research and Practice 32, no. 2 (June 2022): e3222211, 10.17061/phrp3222211. 35702744

[38] Institute P‐COR. Best Practices in Engaging Stakeholders, accessed June 6, 2024, https://research-teams.pcori.org/stakeholders#Planning%20for%20Collaboration.

[39] E. N. Woodward , M. M. Matthieu , U. S. Uchendu , S. Rogal , and J. E. Kirchner , “The Health Equity Implementation Framework: Proposal and Preliminary Study of Hepatitis C Virus Treatment,” Implementation Science 14, no. 1 (2019): 26, 10.1186/s13012-019-0861-y. 30866982 PMC6417278

[40] A. Donabedian , “Evaluating the Quality of Medical Care,” Milbank Quarterly 83, no. 4 (2005): 691–729, 10.1111/j.1468-0009.2005.00397.x. 16279964 PMC2690293

[41] K. P. Weinfurt , A. F. Hernandez , G. D. Coronado , et al., “Pragmatic Clinical Trials Embedded in Healthcare Systems: Generalizable Lessons From the NIH Collaboratory,” BMC Medical Research Methodology 17, no. 1 (September 2017): 144, 10.1186/s12874-017-0420-7. 28923013 PMC5604499

[42] S. Moll , M. Wyndham‐West , G. Mulvale , et al., “Are You Really Doing ‘Codesign’? Critical Reflections When Working With Vulnerable Populations,” BMJ Open 10, no. 11 (November 2020): e038339, 10.1136/bmjopen-2020-038339. PMC7640510 33148733

[43] J. Sumner , C. W. T. Ng , K. E. L. Teo , A. L. T. Peh , and Y. W. Lim , “Co‐Designing Care for Multimorbidity: A Systematic Review,” BMC Medicine 22, no. 1 (February 2024): 58, 10.1186/s12916-024-03263-9. 38321495 PMC10848537

[44] J. McMahon , C. Brown Wilson , L. Hill , et al., “Optimising Quality of Life for People Living With Heart Failure in Care Homes: Protocol for the Co‐Design and Feasibility Testing of a Digital Intervention,” PLoS One 18, no. 7 (2023): e0288433, 10.1371/journal.pone.0288433. 37432917 PMC10335659

[45] J. Langley , R. Wassall , A. Geddis‐Regan , et al., “Putting Guidelines Into Practice: Using Co‐Design to Develop a Complex Intervention Based on NG48 to Enable Care Staff to Provide Daily Oral Care to Older People Living in Care Homes,” Gerodontology 40, no. 1 (March 2023): 112–126, 10.1111/ger.12629. 35288971

[46] R. R. Baier , E. McCreedy , R. Uth , D. R. Gifford , and T. Wetle , “Nursing Home Leaders' Perceptions of a Research Partnership,” Aging Clinical and Experimental Research 33, no. 12 (December 2021): 3371–3377, 10.1007/s40520-021-01847-6. 33811623 PMC8019299

[47] N. F. Douglas , S. Browning , and K. Claypool , “Preliminary Evidence for Dementia Collaborative Coaching,” American Journal of Speech‐Language Pathology 32, no. 5 (September 2023): 2146–2157, 10.1044/2023_ajslp-22-00367. 37437528 PMC10567118

[48] C. L. Chan , M. Taljaard , G. A. Lancaster , J. C. Brehaut , and S. M. Eldridge , “Pilot and Feasibility Studies for Pragmatic Trials Have Unique Considerations and Areas of Uncertainty,” Journal of Clinical Epidemiology 138 (October 2021): 102–114, 10.1016/j.jclinepi.2021.06.029. 34229091 PMC8996744

[49] J. A. Palmer , V. A. Parker , L. R. Barre , et al., “Understanding Implementation Fidelity in a Pragmatic Randomized Clinical Trial in the Nursing Home Setting:A Mixed‐Methods Examination,” Trials 20, no. 1 (November 2019): 656, 10.1186/s13063-019-3725-5. 31779684 PMC6883560

